# Mode of action of plectasin-derived peptides against gas gangrene-associated *Clostridium perfringens* type A

**DOI:** 10.1371/journal.pone.0185215

**Published:** 2017-09-21

**Authors:** Xueling Zheng, Xiumin Wang, Da Teng, Ruoyu Mao, Ya Hao, Na Yang, Lifen Zong, Jianhua Wang

**Affiliations:** 1 Key Laboratory of Feed Biotechnology, Ministry of Agriculture, Beijing, China; 2 Gene Engineering Laboratory, Feed Research Institute, Chinese Academy of Agricultural Sciences, Beijing, China; VIT University, INDIA

## Abstract

NZ2114 and MP1102 are novel plectasin-derived peptides with potent activity against Gram-positive bacteria. The antibacterial characteristics and mechanism of NZ2114 and MP1102 against gas gangrene-associated *Clostridium perfringens* were studied for the first time. The minimal inhibitory concentration and minimal bactericidal concentration of NZ2114 and MP1102 against resistant *C*. *perfringens* type A strain CVCC 46 were 0.91 μM. Based on the fractional inhibitory concentration index (FICI) result, an additive or synergic effect was observed between NZ2114 (FICI = 0.5~0.75) or MP1102 (FICI = 0.375~1.0) and antibiotics. The flow cytometry, scanning and transmission electron microscopy analysis showed that both NZ2114 and MP1102 induced obviously membrane damage, such as the leakage of cellular materials, partial disappearance of the cell membrane and membrane peeling, as well as retracting cytoplasm and ghost cell. The gel retardation and circular dichroism (CD) detection showed that NZ2114 and MP1102 could bind to *C*. *perfringens* genomic DNA and change the DNA conformation. Moreover, NZ2114 also interfered with the double helix and unwind the genomic DNA. The cell cycle analysis showed that *C*. *perfringens* CVCC 46 cells exposed to NZ2114 and MP1102 were arrested at the phase I. These data indicated that both NZ2114 and MP1102 have potential as new antimicrobial agents for gas gangrene infection resulting from resistant *C*. *perfringens*.

## Introduction

An anaerobic Gram-positive bacterium-*Clostridium perfringens* is broadly distributed in our environment, coexisting with foods, sewage water, soils, feces and the normal intestinal microbiota of human and animals [1]. The *C*. *perfringens* strains are subdivided into five toxinotypes (A–E) on the basis of the production of toxins, including α, β, ε and ι [1,2], which leading to a wide range of diseases in humans or livestock, ranging from type A gas gangrene to enteritis syndromes [3]. Type C infections are common in newborn animals due to rapid colonization in intestine, which cause a well-known disease syndrome, but type A infections are now recognized with increasing frequency in neonatal and weaned animals, which result in gas accumulation; approaches to control is both different and more complex than those of type C infections [4]. Herein, gas gangrene is an acute rapidly progressive disease that affects muscle tissue, fascia and skin infection, which characterized by marked tissue destruction, gas production, sepsis, and massive death of tissue [5]. Gas gangrene infection consists of spontaneous and traumatic gangrene and approximately 80% traumatic gangrene is caused by *C*. *perfringens* [5].

Antibiotics play a crucial role in clinical treatment of diseases caused by *C*. *perfringens*. However, misuse or overuse of antibiotics has caused emergence and spread of many multidrug-resistant (MDR) *C*. *perfringens* [6–8]. Many commonly used antibiotics, such as tetracycline, bacitracin, and lincomycin, were found to have a mild or weak antibacterial activity toward pathogenic *C*. *perfringens* [6,9]. Moreover, horizontal diffusion of resistant genes increased by genetic background flow factor of *C*. *perfringens* leads to a rise in MDR strains, which made the therapy of *C*. *perfringens* infections more complicated [10]. In addition, the emergence of MDR bacterium and the ban of antibiotics as growth promoters in the Europe and other counties have resulted in an urgent need to discover novel compounds to combat *C*. *perfringens* infection diseases in the postantibiotic era [11,12].

Plectasin, the first known fungal defensin isolated from *Pseudoplectania nigrella*, can inhibit cell wall synthesis by interacting with the peptidoglycan precursor-Lipid II [13]. Unlike other defensins, plectasin and its analogues-NZ2114, MP1102, and MP1106 have narrow-spectrum antimicrobial activity against Gram-positive bacteria, particularly *Staphylococcus aureus*, *Streptococcus suis*, *Streptococcus pneumoniae*, and *Staphylococcus epidermidis* [14–17], and they are non-hemolytic or non-cytotoxic toward human erythrocytes, epidermal keratinocytes, A549 cells, murine L929 fibroblasts, and porcine intestinal epithelial cell line ZYMSIEC02 [14–21]. In addition, NZ2114, MP1102 and MP1106 showed improved potency and better pharmacokinetic properties in different aspects than its parental peptide-plectasin, including antibacterial activity especially against penicillin- and vancomycin-resistant *S*. *aureus*, *S*. *pneumoniae* and *S*. *suis* strains, the postantibiotic effect, synergistic effect with antibiotics, and stability [15–17]. These findings suggest that plectasin and its derivatives may be attractive candidates for human and animal therapeutic agents. Additionally, our previous study has revealed the mode of action of MP1102 against *C*. *perfringens* type C, which including cell membrane damage, interaction with DNA, and cell cycle arrest in I phase. However, antibacterial characteristics and mode of action of NZ2114 and MP1102 against gas gangrene-related *C*. *perfringens* type A have not yet been elucidated.

In our pre-experiment, the antibiotic sensitivity testing result showed that *C*. *perfringens* type A CVCC 46 can resist multiple antibiotics such as lincomycin, bacitracin, streptomycin, cefotaxime, vancomycin, neomycin, azithromycin, kanamycin, gentamicin, and tetracycline (data not shown). In this study, the antibacterial activity of both NZ2114 and MP1102 toward pathogenic *C*. *perfringens* type A CVCC 46 and their antibacterial action, including disruption of the cell membrane and genomic DNA, were elucidated for the first time, as well as effect on the cell morphology.

## Materials and methods

### Materials

Both NZ2114 and MP1102 were prepared by using the *Pichia pastoris* expression system according to our previous protocols [16,17], with the purities of 94.8% and 96.4%. The resistant *C*. *perfringens* CVCC 46, CVCC 51, and CVCC 1337 strains, which isolated from piglet and rabbit infected gas gangrene, were obtained from the China Veterinary Culture Collection (CVCC). The MDR *C*. *perfringens* strains of JT1, JZ10 and JC2, which isolated from broilers, was graciously provided by Professor Yanfen Jiang, College of Veterinary Medicine, Northwest A & F University.

The bacterial genome DNA extraction kit were supplied from TIANGEN Biotech (Beijing) Co., Ltd. Antibiotics including virginiamycin, aureomycin, bacitracin zinc, lincomycin and vancomycin were obtained from the China Institute of Veterinary Drug Control and Dalian Meilun Biotechand Sangon Biotech (Shanghai) Co., Ltd., respectively. The dye-propidium iodide (PI) was gotten from Sigma-Aldrich (China). All other reagents used meet the need of analytical level.

### Determination of antibacterial activity

The minimum inhibitory and minimal bactericidal concentrations (MICs, MBCs) of NZ2114 or MP1102 toward *C*. *perfringens* strains (CVCC 46, JT1, JZ10, and JC2) were measured by a broth microdilution technique as reported previously [22,23]. Briefly, bacterial cells were anaerobically cultured in nutrient meat broth medium (Beijing Aoboxing Bio-Tech Co. Ltd.) to mid-log phase at 37°C and diluted to 1×10^5^ CFU/mL. Serial twofold dilutions of peptides (10 μL/well) were added into the wells of 96-well microplates, followed by addition of cells suspension (90 μL/well). The plates were incubated anaerobically at 37°C for 12~18 h and the MICs and MBCs were measured according to the pervious described method [24]. All tests were carried out in triplicate.

### Growth kinetics measurement

The time-kill assay was performed to evaluate the in vitro pharmacodynamics of both NZ2114 and MP1102 against *C*. *perfringens* CVCC 46 according to a previous method [25]. Briefly, bacterial cells were cultured in anaerobic meat broth medium overnight and diluted to 1×10^5^ CFU/mL with the same medium. The bacterial cells (5 mL) were transferred into a flask (50 mL size) and followed by the addition of 2×MIC antibacterial drugs. After incubation at 37°C, 100-μL samples were taken from each flask per hour, serially diluted in 0.9% NaCl, and plated to count colonies [26]. The 0.9% NaCl solution was used as a blank control, and conventional antibiotics were served as the positive one. They were repeated three times.

### Synergism assays between NZ2114 or MP1102 and antibiotics

Synergism interaction between NZ2114/MP1102 and antibiotics were measured by using a chequerboard microtiter method [15]. A twofold dilution series of NZ2114, MP1102 and specific antibiotics (from 1/16 to 8×MIC) were added into 96-well microplates in accordance with MIC assay described above. The tests were conducted in triplicate. As described in detail the previous report, the fractional inhibitory concentration index (FICI) refers to the sum of the MIC of each drug when used in combination divided by the drug alone [27]. The data were analyzed by the following equation: FICI = (MIC_drug A_ in combination/MIC_drug A_ alone) + (MIC_drug B_ in combination/MIC_drug B_ alone) [23]. The interaction results were interpreted based on the FICI, as shown in the following: synergy (FICI ≤ 0.5), additivity (0.5 < FICI ≤ 1), indifference (1 < FICI ≤ 4), and antagonism (FICI > 4) as described in a previous study [28].

### Interaction of NZ2114 or MP1102 with the *C*. *perfringens* membrane

#### Membrane permeabilization by flow cytometric analysis

After triple washing with 10 mM PBS (pH 7.4), the mid-logarithmic *C*. *perfringens* cells were diluted to 1×10^8^ CFU/mL and followed by incubation with 1×MIC NZ2114 or MP1102 at 37°C for different times of 5, 30, or 120 min, respectively. Following washing with buffer again, the bacterial cells were mixed with PI (50 μg/mL) at room temperature for 20 min and detected by a FACS Calibur Flow Cytometer (BD, USA) using the CellQuest Pro software (BD, USA) as described previously [29].

#### Electron microscopy observation

Scanning electron microscopy (SEM) is usually used to analyze cell surfaces at high resolution and observe cells morphology [30]. 4×MIC NZ2114 or MP1102 was added into the mid-logarithmic *C*. *perfringens* CVCC 46 (1×10^8^ CFU/mL) cells and incubated for 2 h at 37°C. After centrifugation (1500×g, 5 min), the cells were washed for three times with PBS (0.1 M, pH 7.4) and then fixed in 2.5% glutaraldehyde overnight at 4°C. After washing twice again, the cells were treated using an ethanol series of 50, 70, 85, 95, and 100% (15 min/time), dried by CO_2_, sputtered with gold-palladium, and observed under a QUANTA200 SEM (FEI, Philips, Netherlands) [31].

Similarly, the bacterial cells were mixed with 4×MIC NZ2114 or MP1102, washed, and fixed in 2.5% glutaraldehyde at 4°C overnight according to the above same method. After post-fixation in 1% buffered osmium tetroxide for 2 h, the samples were washed three times with PBS (0.1 M, pH 7.4), treated by the above ethanol series (50–70–85–95–100%), and followed by immerse in acetone and resin solutions. After embedding in Spur resin, the samples were then sectioned, put on Formvar carrier grids and followed by staining with 2% uranyl acetate and lead citrate. The microscopy was performed with a JEM-1400 (JEDL, Japan) [32].

### Interaction of NZ2114 or MP1102 with the bacterial genomic DNA

#### Gel retardation assay

The genomic DNA were extracted from *C*. *perfringens* CVCC 46 with a bacterial DNA kit (TIANGEN, Beijing). The gel retardation experiment was conducted by mixing bacterial DNA with different concentrations of NZ2114 or MP1102 in 20 μL DNA binding buffer (10 mM Tris-HCl (pH 8.0), 5% glycerol, 20 mM KCl, 1 mM dithiothreitol, 1 mM EDTA, and 50 μg/mL BSA) as described previously [25]. The peptide/DNA ratios were 0, 0.5, 1, 2.5, 5.0, and 10.0 (*w/w*), respectively. The peptide/DNA solutions were mixed for 10 min at 37°C and the migration of DNA was analyzed in 0.7% agarose gel by using a Geliance 200 imaging system (PerkinElmer, USA) [31].

#### Circular dichroism (CD) spectroscopy

The CD spectra of genomic DNA extracted from *C*. *perfringens* were measured to examine whether NZ2114 or MP1102 binding cause secondary structure changes in DNA as a previously described method [32]. Both peptides and genomic DNA were mixed at mass ratios of 0, 1.0, 2.5, and 10.0 respectively, incubated for 10 min at room temperature, loaded into a cuvette with 1.0-mm path length and followed by running at 25°C on a CD spectrometer (Pistar π-180, Applied Photophysics, USA). Data are the average of 10 scans with an integration time of 20 s.

#### Cell cycle analysis

The bacterial DNA contents were analyzed by the PI-staining flow cytometry, and the assays were essentially carried out as previous method [33] with some modifications. In brief, 1×MIC peptide solution was added into the mid-log phase *C*. *perfringens* CVCC 46 cells (1×10^8^ CFU/mL) and incubated at 37°C for 0.5 h or 2 h, respectively. After centrifugation for 5 min (1500×g) and washing twice with PBS, the cells were fixed in 75% cool-ethanol (1.8 mL) overnight at 4°C, centrifuged and washed again, and followed by resuspension in 400 μL of PBS (containing 250 μg RNAse A). After incubation for 15 min at room temperature, the cells were stained with 50 μL 0.5 mg/mL PI for 15 min in the dark. Finally, the DNA content and cell cycle phase distribution were determined by a FACS Calibur Flow Cytometer with the ModFit LT software (version 4.1, BD, USA).

## Results

### Antibacterial activity of NZ2114 and MP1102

To evaluate the antimicrobial activity of NZ2114 and MP1102, the MIC and MBC determination were performed against four MDR *C*. *perfringens* strains. The MIC values of NZ2114, MP1102, plectasin, and antibiotics against *C*. *perfringens* CVCC 46, CVCC 51 and CVCC 1137 were 0.91, 0.91, 1.81, and 0.24~8.68 μM, respectively (Table 1), indicating that both peptides have higher antibacterial activity than plectasin and traditional antibiotics in the exception of bacitracin zinc (0.67 μM), virginiamycin (0.24~0.48 μM) or vancomycin (0.69 μM). Additionally, the MBC values of antibiotics and AMPs were identical to or higher than their MIC values. The MIC values of plectasin, NZ2114 and MP1102 against *C*. *perfringens* JT1 were 0.23 or 0.46 μM, equal to or lower than antibiotics (0.46~16.7 μM). Plectasin, NZ2114 and MP1102 displayed the lowest antibacterial activity of against *C*. *perfringens* JZ10 and JC2 with high MIC values (Table 1).

**Table 1:** *In vitro* antibacterial activities of AMPs and antibiotics against MDR *C*. *perfringens* strains.

Antibacterial agents	CVCC 46		CVCC 51	CVCC 1137	JT1	JZ10	JC2
	MIC (μM)	MBC (μM)	MIC (μM)				
**Plectasin**	1.81	1.81	ND	ND	0.23	>14.5	>14.5
**NZ2114**	0.91	0.91	0.91	1.81	0.46	>14.5	>14.5
**MP1102**	0.91	0.91	0.91	1.82	0.46	>14.5	>14.5
**Lincomycin**	8.68	8.68	4.3	2.17	2.2	>277.6	**>**277.6
**Virginiamycin**	0.96	0.96	0.24	0.48	0.46	121.8	60.9
**Bacitracin zinc**	0.67	1.34	1.35	1.35	0.67	>86.1	>86.1
**Vancomycin**	0.69	0.69	0.69	0.69	0.69	22.1	22.1
**Aureomycin**	4.18	8.36	1.04	0.52	16.7	16.7	16.7

ND indicates no detection.

The CVCC 46 strain is resistant to lincomycin, bacitracin and other antibiotics; the CVCC 51 and CVCC 1137 strains are resistant to bacitracin, streptomycin, kanamycin, gentamycin and other antibiotics; the JT1, JC2 and JZ10 strains are resistant to vancomycin, virginiamycin, clindamycin, tetracycline and other antibiotics.

### Growth kinetics measurement

**Fig 1:**
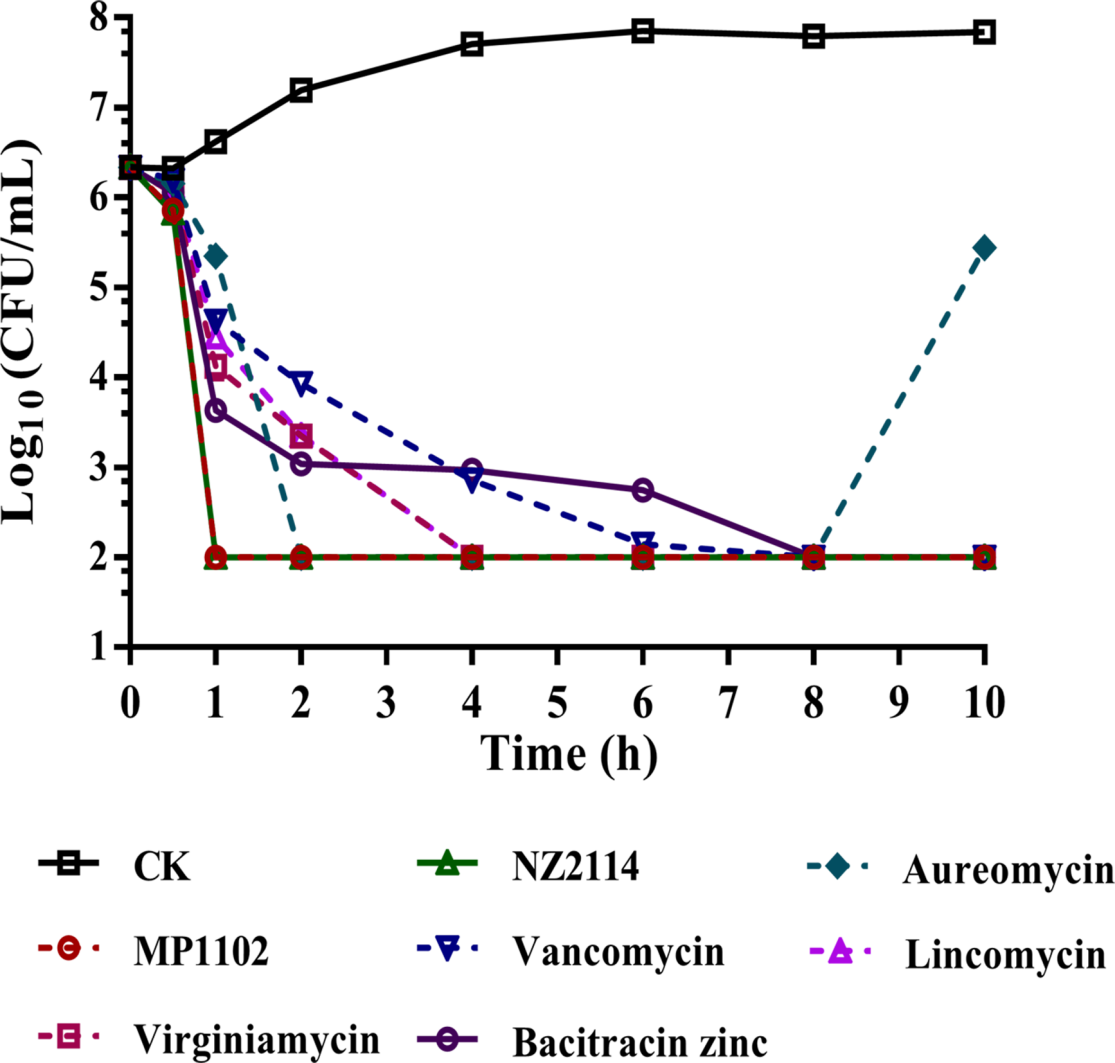
Time-kill curves of NZ2114 and MP1102. Growth kinetic measurements of *C*. *perfringens* exposed to 2×MIC peptide or antibiotics. CK *C*. *perfringens* CVCC 46 were incubated in the presence of medium alone.

The time-kill curve was determined to evaluate the pharmacodynamics of NZ2114 and MP1102 toward MDR *C*. *perfringens* strain CVCC 46. As shown in Fig 1, the bacterial counts (Log10 CFU/mL) steadily increased to 7.8 for *C*. *perfringens* strain in the absence of antibacterial drugs at 6 h, afterwards, which kept nearly constant. After treatment with NZ2114 and MP1102, the bacterial counts have an amazing similarity, which slowly decreased within 0.5 h but sharply decreased half an hour later. Treatments with lincomycin, virginiamycin, and aureomycin led to a 99.9% reduction of bacterial counts within 2~4 h. However, regrowth of *C*. *perfringens* strain CVCC 46 was observed after being treated with aureomycin for 8 h. Additionally, a slightly decrease was be seen in vancomycin and bacitracin zinc treatment groups, which could lead to a 99.9% reduction of bacterial counts until 8 h. Overall, a reduction of bacterial counts of treatment of NZ2114 and MP1102 were more sharply than those of conventional antibiotics treatment at 2×MIC and no bacterial regrowth, compared with treatment with aureomycin, occurred within 10 h.

### Synergism between NZ2114 or MP1102 and antibiotics

As shown in Table 2, all the FICI results showed a synergic or additive effect between NZ2114 and virginiamycin, aureomycin, bacitracin zinc, lincomycin, and vancomycin against MDR *C*. *perfringens* CVCC 46 (FICI = 0.5~0.75). Similarly, an additive or synergic effect was observed between MP1102 and the tested antibiotics against *C*. *perfringens* CVCC 46 (FICI = 0.375~1.0). There were no indifference and antagonism.

**Table 2:** *In vitro* activities of NZ2114 and MP1102 in combination with antibiotics against *C*. *perfringens* CVCC 46.

Combination	Variety	MICa (μM)	MIC_C_ (μM)	FIC	FICI
Bacitracin zinc-NZ2114	Bacitracin zinc	0.67	0.1675	0.25	0.5^a^
	NZ2114	0.91	0.2275	0.25	
Virginiamycin-NZ2114	Virginiamycin	0.96	0.48	0.5	0.75^b^
	NZ2114	0.91	0.2275	0.25	
Lincomycin-NZ2114	Lincomycin	8.68	4.34	0.5	0.625^b^
	NZ2114	0.91	0.11375	0.125	
Aureomycin-NZ2114	Aureomycin	8.36	4.18	0.5	0.625^b^
	NZ2114	0.91	0.11375	0.125	
Vancomycin-NZ2114	Vancomycin	0.69	0.345	0.5	0.75^b^
	NZ2114	0.91	0.455	0.2	
Bacitracin zinc-MP1102	Bacitracin zinc	0.67	0.335	0.5	1.0^b^
	MP1102	1.82	0.91	0.5	
Virginiamycin-MP1102	Virginiamycin	0.96	0.12	0.125	0.375^a^
	MP1102	1.82	0.455	0.25	
Lincomycin-MP1102	Lincomycin	8.68	4.34	0.5	0.625^b^
	MP1102	1.82	0.2275	0.125	
Aureomycin-MP1102	Aureomycin	8.36	2.09	0.25	0.375^a^
	MP1102	1.82	0.2275	0.125	
Vancomycin-MP1102	Vancomycin	0.69	0.345	0.5	1.0^b^
	MP1102	1.82	0.91	0.5	

MICa indicates the MIC of drug used alone; MICc indicates the MIC of drug used in combination.

^a^Synergic effect.

^b^Additive effect.

### Effects of NZ2114 or MP1102 on the cell membrane of *C*. *perfringens*

#### Inner membrane integrity

**Fig 2:**
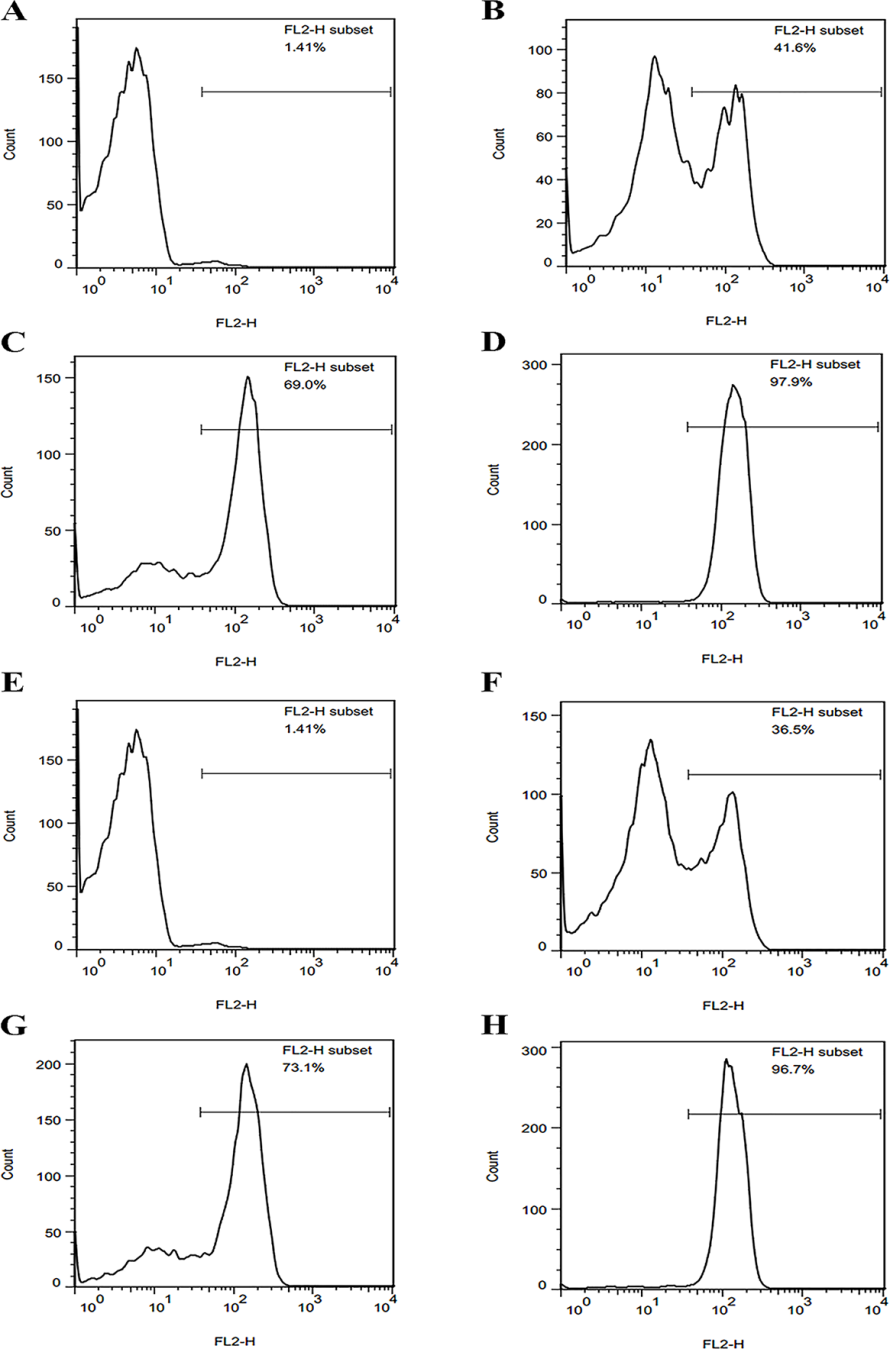
FACScan analysis of PI staining in *C*. *perfringens* CVCC 46 treated with NZ2114 and MP1102. **(A, E)** The untreated *C*. *perfringens* CVCC 46 cells. **(B-D, F-G)** The *C*. *perfringens* CVCC 46 cells treated with 1×MIC NZ2114 **(B-D)** and MP1102 **(F-G)** for 5 **(B, F)**, 30 **(C, G)**, and 120 min **(D, H)**, respectively.

After treatment with NZ2114 or MP1102, the membrane damage of *C*. *perfringens* cells was assayed by staining with PI-dye and then analyzed with a flow cytometer. 2The PI-permeated percentages of cells treated with 1×MIC NZ2114 for 5, 30, or 120 min were 41.6%, 69.0%, or 97.9%, respectively (Fig 2B and 2C) and those treated with 1×MIC MP1102 were 36.5% (5 min), 73.1% (30 min), and 96.7% (120 min), respectively (Fig 2F and 2G), which were much higher than those of the untreated cells (1.41%) (Fig 2A and 2E). This result indicated that the inner membrane of *C*. *perfringens* cells can be disrupted by both NZ2114 and MP1102.

#### SEM observations

**Fig 3:**
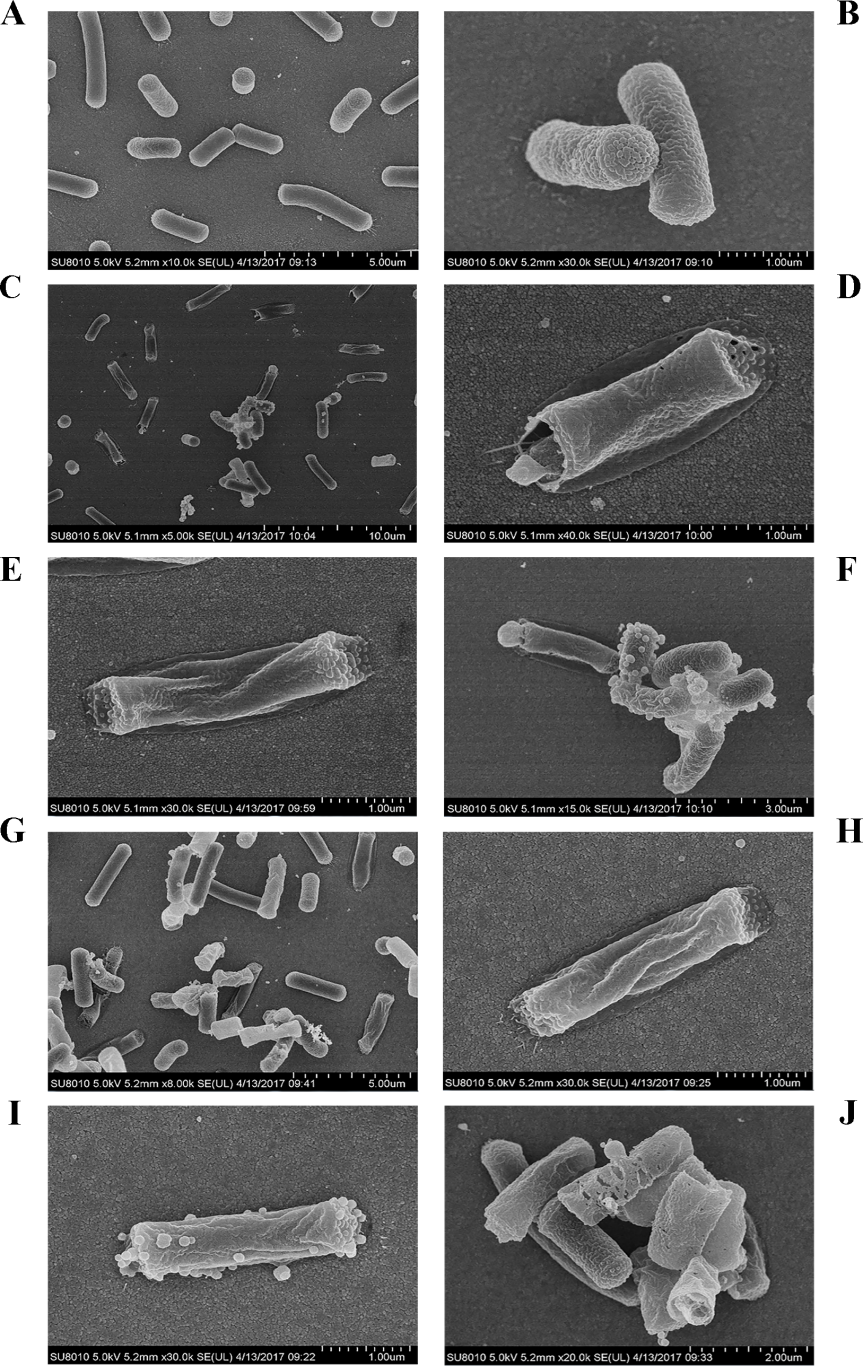
Scanning electron micrographs of *C*. *perfringens* CVCC 46 cells treated with NZ2114 and MP1102. **(A, B)** The untreated *C*. *perfringens* CVCC 46 cells. **(C-J)** The *C*. *perfringens* CVCC 46 cells treated with 4×MIC NZ2114 **(C-F)** and MP1102 **(G-J)** for 2 h, respectively.

SEM was used to directly observe the effects of NZ2114 or MP1102 on the cell morphology and integrity of MDR *C*. *perfringens* CVCC 46. As shown in Fig 3, it was observed the normal intact cell morphology; there was no any cellular disruption or release of intracellular content in the untreated control group. However, after treatment with NZ2114 (Fig 3C–3F) or MP1102 (Fig 3G–3J), the cells exhibited obviously membrane damage (approximately 50%), such as membrane hole, peeling and lysis, the leakage of cellular materials, and retracting cytoplasm.

#### TEM observations

**Fig 4:**
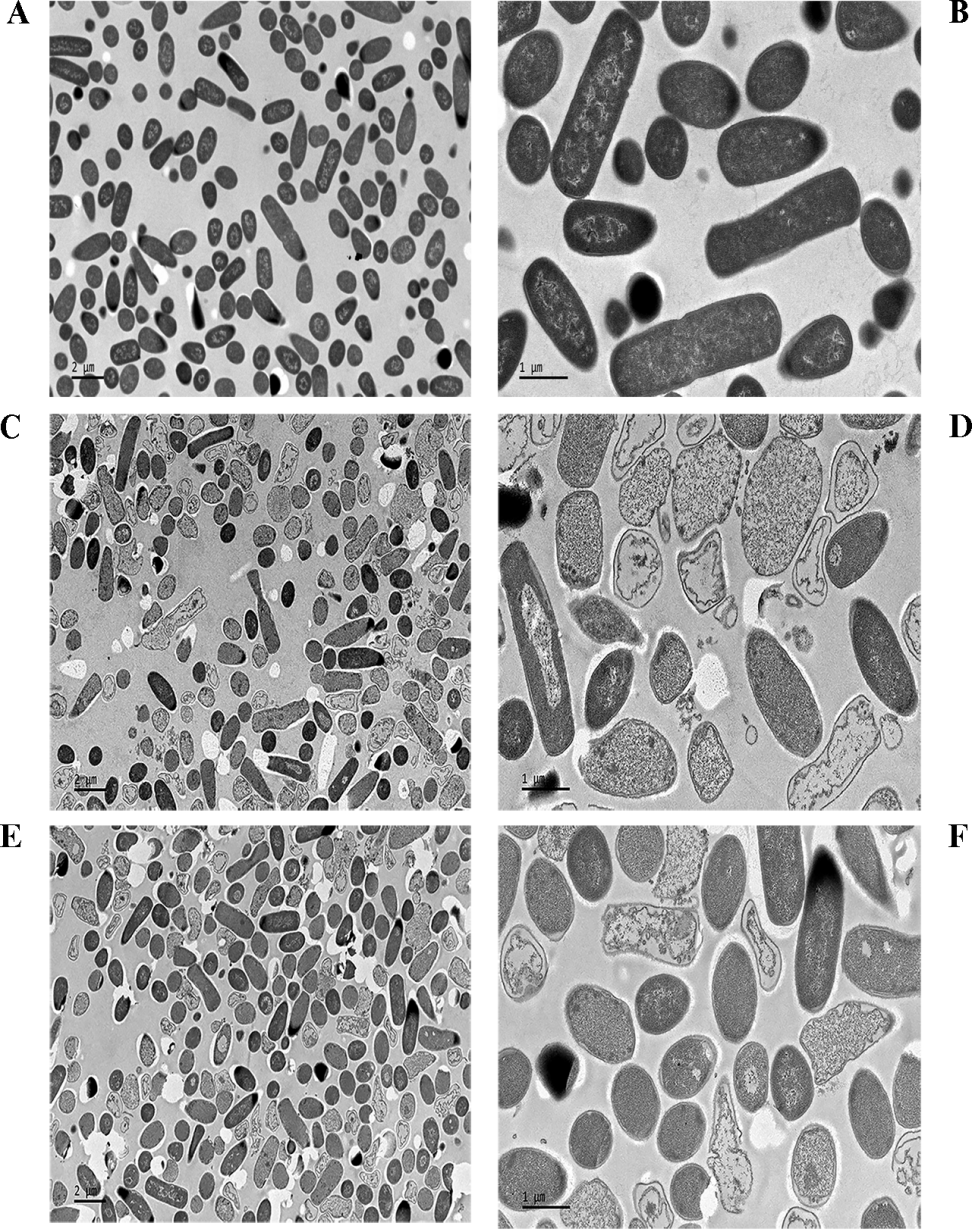
Transmission electron micrographs of *C*. *perfringens* CVCC 46 cells treated with NZ2114 and MP1102. **(A, B)** The untreated *C*. *perfringens* CVCC 46 cells. **(C-F)** The *C*. *perfringens* CVCC 46 cells treated with 4×MIC NZ2114 **(C, D)** and MP1102 **(E, F)** for 2 h, respectively.

After treatment with NZ2114 and MP1102, the cell morphology and intracellular changes were further detected by using TEM. The untreated *C*. *perfringens* cells had intact shapes and no damage was observed in membrane structure. Homogeneous electron density was observed in the cytoplasm (Fig 4A and 4B). However, after treatment with NZ2114 or MP1102, the morphology of the cells appeared deformed and a heterogeneous electron density was observed in the cytoplasm, and (Fig 4D and 4F). It was also observed that the cell disruption, partial disappearance of the cell membrane, and the leakage of cellular material was occurred, which was consistent with the above SEM observation. In addition, retracting cytoplasm and ghost cell were observed in the peptide-treated cells.

### Interaction of NZ2114 or MP1102 with the *C*. *perfringens* cellular DNA

#### DNA gel retardation

**Fig 5:**
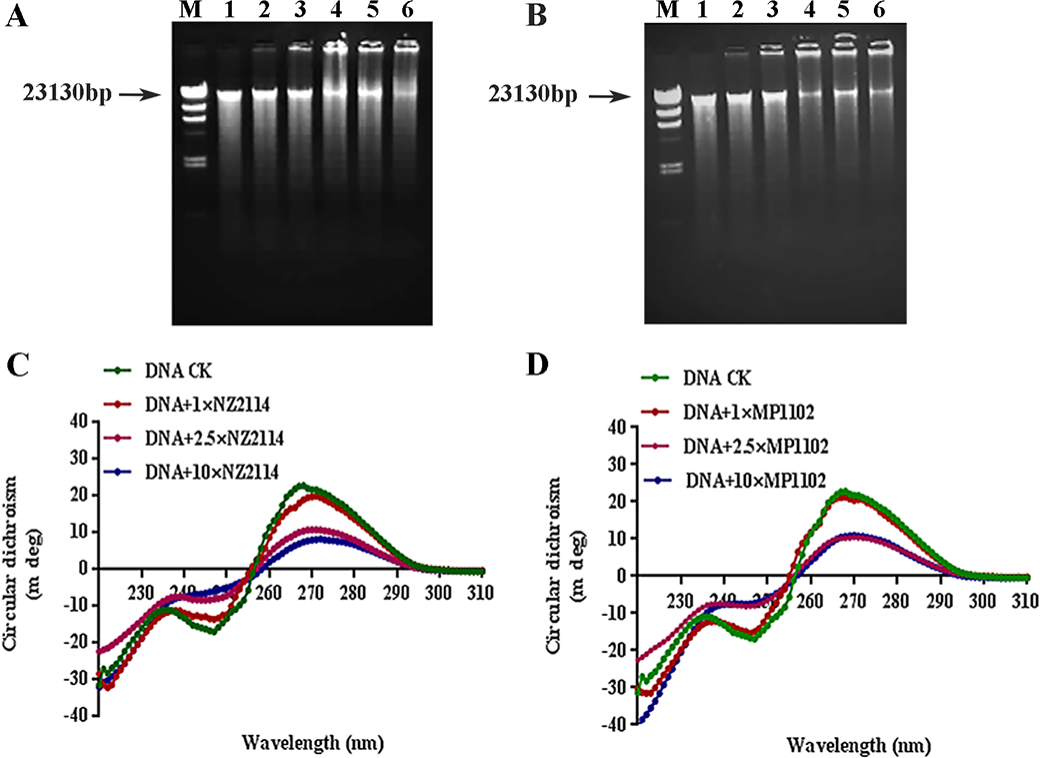
*In vitro* binding of NZ2114 and MP1102 to bacterial genomic DNA. **(A, B)** Gel retardation analysis of the binding of NZ2114 **(A)** and MP1102 **(B)** to genomic DNA. M: DNA Marker λDNA/*Hin*dIII. Lanes 1–6: genomic DNA from *C*. *perfringens* CVCC 46. The mass ratios of peptide and genomic DNA were 10, 5, 2.5, 1, 0.5, and 0, respectively. **(C, D)** CD spectra of genomic DNA from *C*. *perfringens* in the presence of NZ2114 **(C)** and MP1102 **(D)**. The mass ratios of peptide to DNA were 0, 1.0, 2.5, and 10.0, respectively.

The electrophoretic gel mobility shift assay was used to evaluate the DNA-binding capability of peptide to bacterial genomic DNA. As shown in Fig 5A and 5B, NZ2114 and MP1102 interacted with MDR *C*. *perfringens* CVCC 46 genomic DNA. Nearly all DNA moved into the gel at a peptide/DNA mass ratio of 0.5, but the complete retardation in DNA-peptide migration did not appear even at a mass ratio of 10.0. The interaction of NZ2114 and MP1102 with DNA was also verified by the next CD spectroscopy.

#### CD spectroscopy

The CD spectrum is usually used to monitor changes in DNA morphology when drugs interact with DNA [34]. The affinity of NZ2114 and MP1102 binding to DNA were further detected using a CD spectrometer. It appeared a positive peak at approximately 270 nm and a negative one at about 245 nm in the CD spectrum of *C*. *perfringens* genomic DNA (Fig 5C and 5D). After treatment with NZ2114 or MP1102, the DNA ellipticity intensity decreased, indicating the negative correlation with the peptide content. This suggested that NZ2114 and MP1102 maybe bind to *C*. *perfringens* genomic DNA which changing the DNA conformation. There is a significant difference between NZ2114 and MP1102. The slight redshift was observed in NZ2114, indicating that NZ2114 interfere with the double helix and unwind the genomic DNA. These results further illustrated that NZ2114 and MP1102 could interact with *C*. *perfringens* DNA.

#### Cell cycle analysis

**Fig 6:**
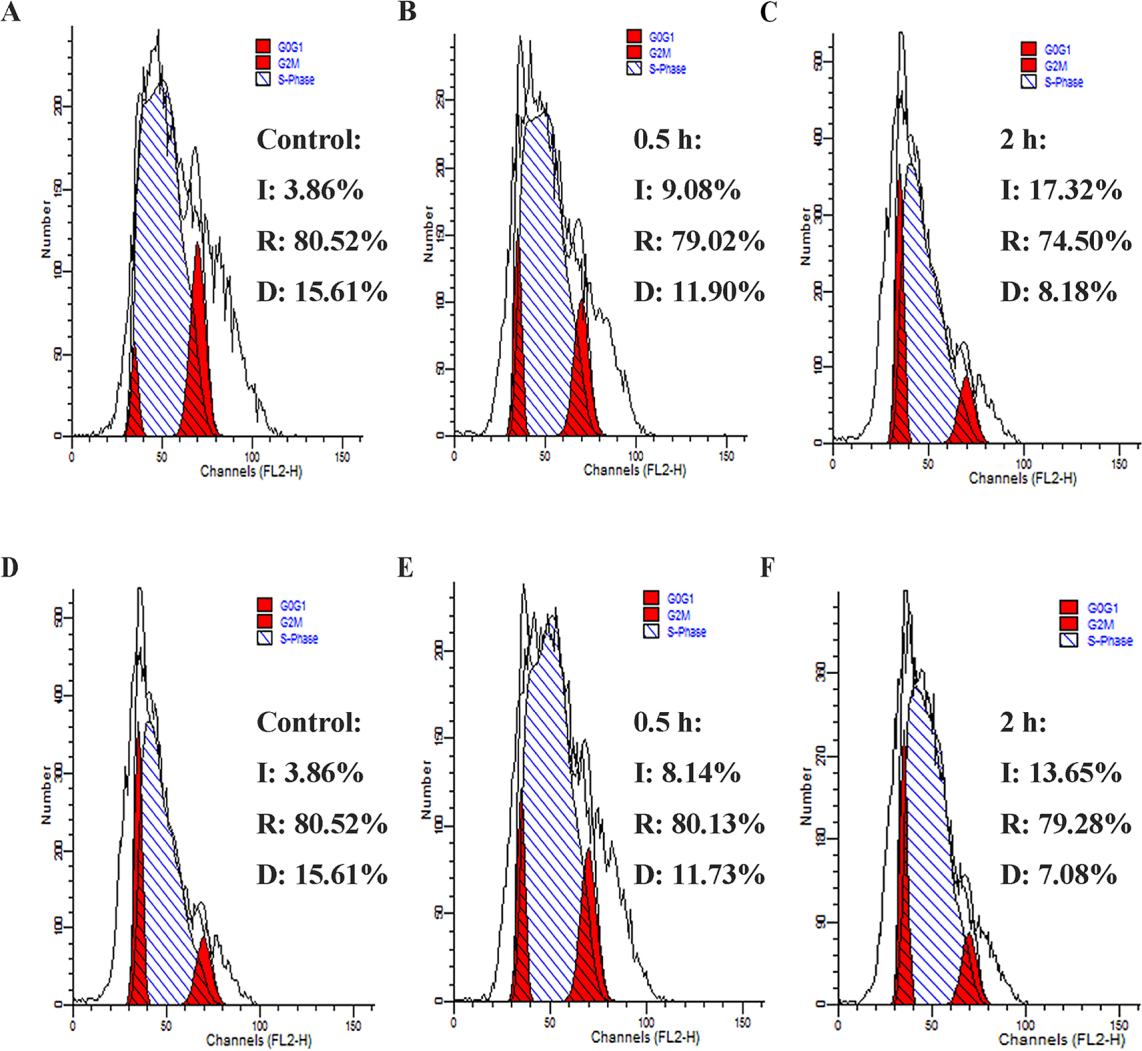
FACScan analysis of the cell cycle of *C*. *perfringens* CVCC 46 cells treated with NZ2114 and MP1102. **(A, D)** The untreated *C*. *perfringens* CVCC 46 cells. **(B, C, E, F)** The cells treated with 1×MIC NZ2114 **(B, C)** and MP1102 **(E, F)** for 0.5 h **(B, E)** and 2 h **(C, F)**, respectively.

The cell cycle of prokaryotes has three phase: the initiation (I), replication (R) and division (D). Once the bacterial DNA is disrupted, cell cycle progression will be inhibited and cell division can’t move into the next phase, causing cell cycle to remain in either phase [32]. After treatment with 1×MIC NZ2114 or MP1102 for 0.5 h and 2 h, *C*. *perfringens* CVCC 46 cell were detected by flow cytometry to analyze their effect on cell division. As showed in Fig 6, the ratios of the normal cells in I, R, and D phases were 3.86%, 80.52% and 15.61%, respectively. The ratio of *C*. *perfringens* cells treated with NZ2114 in phase I increased significantly, which ranging from 9.08% to 17.32%, while it decreased in phase R and D. Exposure to MP1102, the ratio of cells increased significantly from 8.14% to 13.65% in phase I, but decreased in phase R and D. These results indicated both MP1102 and NZ2114 induced the cell cycle of *C*. *perfringens* arrested at I phase and seriously disturbed initiation of the cell cycle.

## Discussion

Pathogenic *C*. *perfringens* acts as an important role in causing intestinal and histotoxic infections in humans and animals [2]. Over a long period in the past, antibiotics have been performed a vital status in curing disease caused by *C*. *perfringens*. However, with the emergence of many antibiotics resistance in *C*. *perfringens*, scientists have paid more attentions to search for the newly effective alternatives to antibiotic usage [6–8]. Antimicrobial peptides (AMPs) are distributed in all living organisms as part of the host innate immunity [35] and they have rapidly become the promising therapeutic agents due to their potency and modes of action, including targeting the cell wall [13], cell membrane [36], or cytoplasm [37,38], interaction with DNA, and induction of the apoptosis-like cell death [32]. It has been demonstrated that NZ2114 and MP1102 display significant activity against Gram-positive bacteria, but not Gram-negative bacteria. Similarly to plectasin, NZ2114 and MP1102 may directly bind to Lipid II precursor of Gram-positive bacterial cell wall, which don’t occur in Gram-negative bacteria due to the large size (approximately 4.4 kDa) of both peptides [15,16], inhibiting the passage across the outer membrane [39,40].

Both NZ2114 and MP1102 have improved antibacterial activity against *S*. *aureus*, especially MRSA and MSSA strains, compared to their parental peptide-plectasin [15,16]; the same result of antibacterial activity of the two peptides against MDR *C*. *perfringens* was obtained in this study (Table 1). The bactericidal tendency of NZ2114 and MP1102 showed they could lead to a 99.9% reduction of bacterial counts within 1 h (Fig 1). The characteristics of high bactericidal efficiency, low toxicity and no resistance of NZ2114 and MP1102 make them very attractive for future work.

Clinical monotherapy against *C*. *perfringens* is limited by the increasing severity of antibiotics’ resistance [9,41]. The results of antibacterial activities of NZ2114 or MP1102 combined with traditional antibiotics against MDR *C*. *perfringens* CVCC 46 showed a synergic or additive effect between NZ2114 (FICI = 0.5~0.75) or MP1102 (FICI = 0.375~1.0) and virginiamycin, aureomycin, bacitracin zinc, lincomycin, and vancomycin (Table 2), which may improve antibacterial activity and help to reduce the amount of conventional antibiotics use to inhibit or delay the occurrence of bacterial resistance to antibiotics.

Most AMPs can disturb the cell membrane, which leading to morphological change in the membrane structure [23,32,42]. To determine whether both NZ2114 and MP1102 have an effect on the cell membrane, a membrane permeability assay was firstly performed using FACS. The PI dye is commonly used as a viability intracellular marker that can enter into the impaired cells and insert into DNA. The result of PI influx into *C*. *perfringens* cells suggested that NZ2114 and MP1102 induced cytoplasmic membrane damage (Fig 3). Additionally, after treatment with 1×MIC NZ2114 and MP1102 for 5–120 min, the PI-stained percentages of *C*. *perfringens* type A CVCC 46 cells were 41.6~97.9% and 36.5~96.7%, which were markedly higher than those of MP1102 against *C*. *perfringens* type C CVCC 61 cells (3.11~5.67%) in our previous study [23]. This result indicated that MP1102 maybe have a different mode of action against *C*. *perfringens* type A and against type C.

To further investigate the interaction between the two peptides and cell membrane, we tested the effects of NZ2114 and MP1102 on *C*. *perfringens* cells morphology by SEM and TEM. After treatment with NZ2114 and MP1102, obvious cell membrane damage of *C*. *perfringens* cells was found in SEM and TEM observation, such as the partial disappearance of the cell membrane, leakage of cellular materials, and membrane peeling (Fig 3 and Fig 4), which was similar to *C*. *perfringens* type C CVCC 61 cells treated with MP1102 [23]. However, the phenomenon of retracting cytoplasm was not observed in the SEM of *C*. *perfringens* type C CVCC 61 cells treated with the MP1102. The morphology and intracellular alterations in cells were also observed, such as cell disruption, retracting cytoplasm, and ghost cell (Fig 4), which is consistent with those of *C*. *perfringens* ATCC 12915 cells treated with synthetic β*-*defensin Gallinacin-6, but with the exception of irregular septum formation in dividing cells [43]. This may be due to different *C*. *perfringens* strain or peptide. Combining with the assays and observations of FACS, SEM, and TEM of MP1102 or NZ2114-treated bacteria, the results suggested that bacterial membranes was an important target of NZ2114 and MP1102.

Three residues substitution of NZ2114 (N9Q, L13V, and R14K) resulted in an increase in the α-helix content (16.7%→33.3%) and hydrophobicity (0.48→0.56) of MP1102, but their positive charge and isoelectric point had no difference [16]. It is generally considered that the higher α-helix index and hydrophobic moment of AMPs help to increase the ability of transmembrane and the interaction with the membrane [44–46]. However, in this study, no difference was found in the results of FACS, SEM, and TEM between MP1102 and NZ2114-treated *C*. *perfringens* CVCC 46 cells.

More and more evidence showed that cell membrane is not the only way to kill pathogenic microorganisms, which suggests other potential intracellular targets and different mechanisms of killing bacteria [25,32,33]. Previous studies have proved that AMPs can also bind intracellular macromolecules and inhibit their synthesis and biological functions after they traverse the cell membrane and enter into the cytoplasm [31,33]. In this study, gel retardation and CD experiments suggested that NZ2114 and MP1102 can interact with the genomic DNA of *C*. *perfringens* CVCC 46 by changing the DNA conformation (Fig 5). In addition, NZ2114 also interfered with the double helix and undid the genomic DNA (Fig 5C).

The interaction of AMPs and DNA may lead to disturb gene expression, transcription, and protein expression, which effectively shuts down or blocks the synthesis of macromolecules such as protein and receptor synthesis, causing the disruption of the bacterial life substances that results in cells death [33]. The cell cycle analysis showed that NZ2114 or MP1102-treated *C*. *perfringens* CVCC 46 cells were arrested at the phase I (Fig 6), which is consistent with that of *C*. *perfringens* CVCC 61 cells exposed to MP1102 [23]. Taken together with the gel retardation and CD, all these data indicated that DNA may be another target of MP1102 and NZ2114 and the binding affinity of the two peptides to genomic DNA may contribute to their antibacterial activity toward *C*. *perfringens*.

These results conferred a novel evidence of the antimicrobial action of NZ2114 and MP1102 toward MDR *C*. *perfringens* type A CVCC 46, which will lay the theoretic foundation for the application of NZ2114 and MP1102 as novel effective alternatives to antibiotics against gas gangrene-related *C*. *perfringens*.

In conclusion, MP1102 and NZ2114 showed the good antibacterial activity and a synergic or additive effect combining with virginiamycin, aureomycin, bacitracin zinc, lincomycin, and vancomycin. The antibacterial efficacy of the two peptides is ascribed to the cell membrane damage. Both NZ2114 and MP1102 can penetrate impaired membrane barrier, interfere DNA synthesis by altering DNA conformation and lead to the cell cycle arrest. Generally, the mode of action is related to destruction of the bacterial cell membrane and interference with bacterial DNA. Therefore, both NZ2114 and MP1102 have the potency to be developed as new candidates of antibacterial agents in the fight against gas gangrene infection caused by *C*. *perfringens*.
